# Editorial: Dietary modulation of gut microbiota-X axis

**DOI:** 10.3389/fnut.2025.1693629

**Published:** 2025-09-23

**Authors:** Bowen Li, Francesca Bottacini, Yuhui Yang

**Affiliations:** ^1^College of Food Science, Southwest University, Chongqing, China; ^2^Department of Biological Sciences, Munster Technological University, Cork, Ireland; ^3^College of Food Science and Engineering, Henan University of Technology, Zhengzhou, China

**Keywords:** gut microbiota-X axis, gut, dietary, microbiota, health

## 1 The gut microbiota-X axis: an integrative physiological framework

The concept of the “Gut Microbiota-X axis” signifies a fundamental shift in our understanding of how diet systemically influences host physiology via microbial mediation (1). This axis embodies a dynamic, multidirectional communication network in which gut commensals metabolize nutritional substrates into bioactive compounds—such as vitamins, neurotransmitters, and immunomodulators—that directly engage with extra-intestinal organs (2). Accumulating evidence establishes gut microbiota as key metabolic interpreters, translating dietary intake into molecular signals that modulate the neuro-endocrine-immune network (3).

Central to this paradigm is the role of the gut microbiota—a diverse ecosystem of bacteria, archaea, viruses, and fungi—which acts as a metabolic interface, converting dietary and host-derived compounds into bioactive molecules. Notable among these are microbiota metabolites like short-chain fatty acids (SCFAs; e.g., butyrate, propionate, and acetate), produced from dietary fiber fermentation, which function as epigenetic regulators through histone deacetylase (HDAC) inhibition (4). Similarly, microbial transformation of primary bile acids into secondary bile acids activates nuclear receptors such as FXR and TGR5, thereby modulating metabolic and inflammatory pathways systemically (5).

The immune system serves as a major conduit for this cross-organ communication. Microbial components and metabolites continuously interact with gut-associated lymphoid tissue (GALT), shaping immune cell differentiation (e.g., of regulatory T cells) and cytokine production, which in turn exert distal effects on organ inflammation and functionality (6). Additionally, the vagus nerve provides a direct neural pathway through which gut signals influence central nervous system activity, while endocrine mechanisms involving gut hormones such as peptide YY (PYY) and glucagon-like peptide-1 (GLP-1) further integrate metabolic and cognitive functions (7). Dysbiosis—a disruption of microbial homeostasis—compromises this intricate exchange, often increasing intestinal permeability and permitting leakage of microbial products like lipopolysaccharide (LPS) into circulation (8). This can initiate a state of chronic low-grade inflammation, contributing to the pathophysiology of multiple diseases across organ systems, including Alzheimer's disease (via the gut-brain axis) (9), non-alcoholic fatty liver disease (via the gut-liver axis) (10), and psoriasis (via the gut-skin axis) (11). Thus, the gut microbiota-X axis functions as a critical physiological integrator, wherein diet-microbiota interactions fine-tune systemic homeostasis and organismal resilience.

## 2 Thematic advancements: mechanistic elucidation and clinical translation

This Research Topic compiles cutting-edge research exploring the intricate bidirectional relationship between dietary components and the gut microbiota-X axis, emphasizing its profound implications for host health and disease pathogenesis. The collected studies employ diverse methodologies—ranging from preclinical animal models and randomized controlled trials (RCTs) to large-scale epidemiological analyses and systematic reviews—to elucidate specific mechanisms and therapeutic potentials. A prominent theme is the targeted use of probiotics to modulate gut microbial composition and function for health benefits. Key findings demonstrate the efficacy of specific strains, such as *Bifidobacterium adolescentis* CCFM1447 in mitigating osteoporosis by enriching intestinal bacteria capable of vitamin D conversion (Yu, Tian et al.), and *Bifidobacterium longum* subsp. *infantis* CCFM1426 enhances the anti-colitic effects of vitamin A through retinoic acid restoration and microbiota modulation in murine models of ulcerative colitis (Yu, Huang et al.). The translational potential of probiotics is further supported by an RCT showing specific formulations (*Lactobacillus acidophilus, Lactobacillus casei, Lactobacillus rhamnosus*; 3 × 10^9^ CFU) effectively mitigate stress and inflammation in malnourished adults via gut microbiota modulation (Ahmadi-Khorram et al.). Beyond live bacteria, the issue explores broader dietary strategies, including granola with multiple prebiotics increasing *Bifidobacterium* abundance and improving stress/sleepiness in Japanese participants (Sasaki et al.), and the therapeutic application of fecal microbiota transplantation (FMT) in severe food intolerance (Huang, Huang et al.).

A second major focus involves the development and application of novel indices to quantify the relationship between diet, gut microbiota, and disease risk in human populations. Several studies leverage the US National Health and Nutrition Examination Survey (NHANES) data to establish significant associations between a proposed “dietary index for gut microbiota” and specific pathologies. Zhang X. et al. link this index to female infertility, while Wang et al. demonstrate its correlation with metabolic dysfunction-associated steatotic liver disease (MASLD). Most notably, Huang, Liu et al. establish an association between this dietary index and diabetes, proposing phenotypic age and body mass index (BMI) as significant mediators in this relationship, highlighting the complex interplay of diet, microbes, metabolic health, and aging. The critical role of microbial metabolites beyond vitamins is underscored in a systematic review and meta-analysis by Xie L. et al., which synthesizes preclinical evidence supporting the therapeutic potential of microbially-derived short-chain fatty acids (SCFAs) for acute lung injury. Furthermore, a review by Zhang R. et al. examines the intricate links between dietary habits, the gut-brain axis, and cognitive function, emphasizing the microbiota's role in neurobehavioral outcomes. Supporting this, Ding et al. present evidence suggesting early childhood probiotic intake may modify the association between prenatal micronutrient supplementation and neurobehavioral development in preschoolers. Finally, Xie Q. et al. review the emerging therapeutic potential of probiotics and postbiotics in targeting apoptosis for the treatment and prevention of digestive diseases. Collectively, this Research Topic significantly advances our understanding of how dietary components—from specific foods and supplements to broader dietary patterns—can be strategically manipulated to shape gut microbial communities and their functional outputs (vitamin conversion, SCFA production, immune modulation, neuroactive metabolite generation), thereby offering novel avenues for preventing and treating a wide spectrum of conditions including metabolic diseases, gastrointestinal disorders, osteoporosis, neurological impairments, and inflammatory states. The research highlights the move toward personalized nutrition and microbiota-targeted therapeutics while also identifying key areas for future investigation, such as refining dietary indices, elucidating strain-specific effects, and translating preclinical findings into robust clinical applications.

## 3 Future insights

Future research on the dietary modulation of the gut microbiota-X axis will likely focus on precision nutrition strategies that account for individual variability in microbiome composition, host genetics, and environmental factors. A key direction involves the development of targeted interventions using specific dietary components—such as prebiotics, polyphenols, and fermented foods—to selectively modulate microbial taxa and metabolic pathways that influence the X axis (e.g., brain, liver, or bone). Advanced multi-omics integration, including metagenomics, metabolomics, and transcriptomics, will be essential to elucidate mechanistic links between diet, microbial metabolites (e.g., SCFAs, bile acids, and tryptophan derivatives), and host physiological outcomes. Furthermore, long-term randomized controlled trials and longitudinal studies are needed to establish causal relationships and evaluate the sustainability of dietary interventions in improving health outcomes related to the microbiota-X axis.

Another promising area is the application of artificial intelligence and machine learning to analyze complex datasets and predict individual responses to dietary interventions. This approach could enable the design of personalized dietary recommendations that dynamically adapt to shifts in an individual's gut microbiome and health status. Additionally, there is growing interest in exploring the role of chrono nutrition and meal timing in modulating microbial rhythms and their interactions with host circadian biology. Future studies should also investigate the synergistic effects of diet and other lifestyle factors, such as exercise and stress management, on the microbiota-X axis. Ultimately, translating these insights into clinical practice and public health nutrition will require interdisciplinary collaboration and the development of functional foods or nutraceuticals that are evidence-based, effective, and accessible.
